# Canaliculitis: Are we missing the diagnosis?

**DOI:** 10.4103/0974-620X.57318

**Published:** 2009

**Authors:** Abdullah Al-Mujaini, Upender Wali, Rana Al-Senawi

**Affiliations:** Department of Ophthalmology, Sultan Qaboos University Hospital, Muscat, Sultanate of Oman

**Figure 1:**
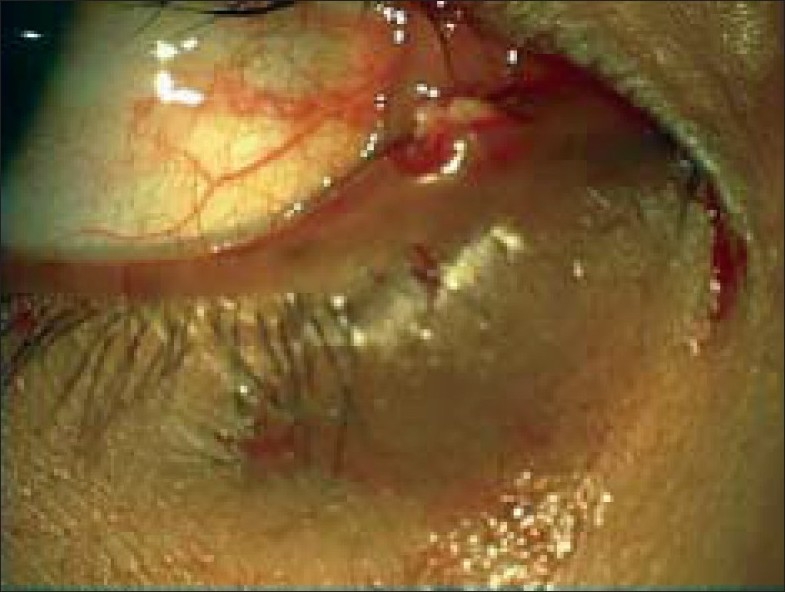
OD: lower canalicular swelling and pouting of the punctum

**Figure 2:**
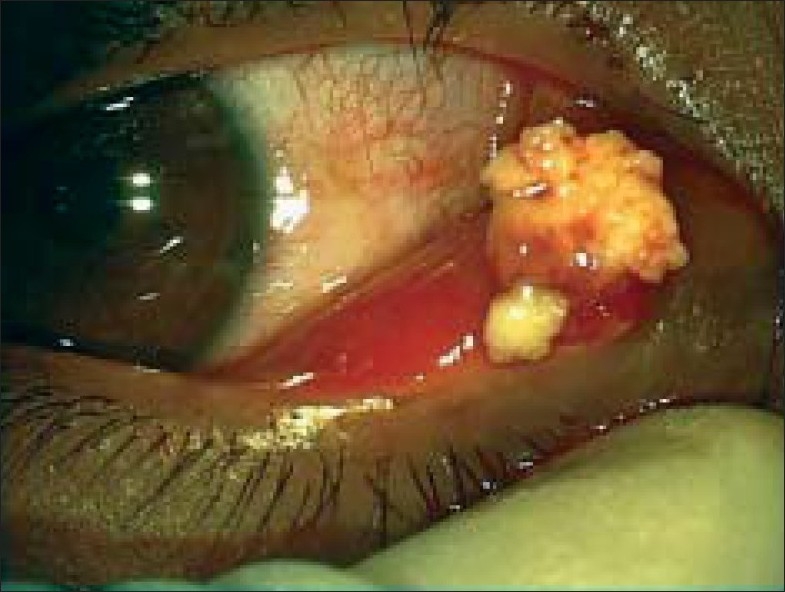
OD: Intraoperative expression of concretions.

A 22-year-old female patient presented for further evaluation and second opinion of chronic, long standing tearing, discharge and swelling of right lower lid since three years. She had been diagnosed and treated in other hospitals for naso-lacrimal duct obstruction and advised to go for dacryocystorhinostomy. Examination showed best corrected visual acuity of 20/20 in both eyes. Right eye revealed lower canalicular swelling and pouting of the punctum [Figure 1]. On gentle pressure over the lacrimal sac there was regurgitation of mucoid discharge through the lower punctum. Syringing was patent through the lower canaliculus. A clinical diagnosis of chronic canaliculitis was made and the patient had three snip procedure combined with canaliculotomy. Large amount of concretions were expressed intraoperatively [Figure 2] and sent for histopathological examination, which was reported as an inflammatory granulation tissue with exudates containing branching filamentous structures positive for Gommori Methanamine Silver stain. Gram stain and PAS studies were indicative of *Actinomyces*.

Primary chronic canaliculitis is an uncommon disease of the proximal lacrimal system that can be overlooked in terms of misdiagnosis and inappropriate management.[1] It might present as a case of epiphora, chronic mucous discharge or persistent, long standing conjunctivitis that is refractory to any modality of therapy. As the block of the canaliculi is incomplete, partial syringing of the passages is possible, differentiating this condition from nasolacrimal duct obstruction secondary to any other etiology.

*Actinomyces israelii* is the most common causative agent encountered in canaliculitis, but other bacteria (*Fusobacterium* and *Nocardia* species), fungi (*Candida albicans, Fusarium* and *Aspergillus* species) and viruses (herpes simplex, varicella zoster) should also be considered as a cause.[1]

The differential diagnosis of chronic canaliculitis includes; migration of silicone punctal plug, chronic conjunctivitis or even rarely carcinoma of the lacrimal canaliculus.[2]

Although very few literature reports showed that intracanalicular irrigation with broad spectrum antibiotics may obviate the need for surgical management in treating chronic canaliculitis, canalicular debridement in the form of canaliculotomy and expression of all concretions is still the mainstay of treatment and more effective than conservative management.[3,4]
